# Minimally invasive third ventriculostomy with stereotactic internal shunt placement for the treatment of tumor-associated noncommunicating hydrocephalus

**DOI:** 10.1007/s00701-023-05768-3

**Published:** 2023-09-07

**Authors:** Sebastian Niedermeyer, Nicole A. Terpolilli, Pia Nerlinger, Jonathan Weller, Michael Schmutzer, Stefanie Quach, Niklas Thon

**Affiliations:** Department of Neurosurgery, LMU Hospital, Ludwig-Maximilian-University Munich, Marchioninistrasse 15, 81377 Munich, Germany

**Keywords:** Stereotactic ventriculostomy, Minimally invasive, Obstructive hydrocephalus

## Abstract

**Background:**

Intracranial tumors can cause obstructive hydrocephalus (OH). Most often, symptomatic treatment is pursued through ventriculoperitoneal shunt (VS) or endoscopic third ventriculostomy (ETV). In this study, we propose stereotactic third ventriculostomy with internal shunt placement (sTVIP) as an alternative treatment option and assess its safety and efficacy.

**Methods:**

In this single-center, retrospective analysis, clinical symptoms, procedure-related complications, and revision-free survival of all patients with OH due to tumor formations treated by sTVIP between January 2010 and December 2021 were evaluated.

**Results:**

Clinical records of thirty-eight patients (11 female, 27 male) with a mean age of 40 years (range 5–88) were analyzed. OH was predominantly (in 92% of patients) caused by primary brain tumors (with exception of 3 cases with metastases). Following sTVIP, 74.2% of patients experienced symptomatic improvement. Preoperative headache was a significant predictor of postoperative symptomatic improvement (OR 26.25; 95% CI 4.1–521.1; *p* = 0.0036). Asymptomatic hemorrhage was detected along the stereotactic trajectory in 2 cases (5.3%). One patient required local revision due to CSF fistula (2.6%); another patient had to undergo secondary surgery to connect the catheter to a valve/abdominal catheter due to CSF malabsorption. However, in the remaining 37 patients, shunt independence was maintained during a median follow-up period of 12 months (IQR 3–32 months). No surgery-related mortality was observed.

**Conclusions:**

sTVIP led to a significant symptom control and was associated with low operative morbidity, along with a high rate of ventriculoperitoneal shunt independency during the follow-up period. Therefore, sTVIP constitutes a highly effective and minimally invasive treatment option for tumor-associated obstructive hydrocephalus, even in cases with a narrow prepontine interval.

## Introduction

In adults, newly diagnosed OH is most commonly caused by periventricular or periaqueductal tumor formation [2]. Ventriculoperitoneal shunt (VS) or endoscopic third ventriculostomy (ETV) is an established treatment strategy for OH. Although VS remains the gold standard for patients with hydrocephalus, it is associated with a relevant complication and revision rate of 20–30% [3, 21, 23, 27, 36]. This is particularly noteworthy in oncologic patients, especially those with limited life expectancy, as quality of life may be significantly affected by long and/or repeated hospitalizations [3, 21]. Because of the lower morbidity, reduced surgery time and hospitalization, and lower long-term complication rate, ETV is now advised as primary treatment for OH [2, 13]. Factors determining eligibility, success rate, and perioperative risk for ETV are the configuration of the third ventricle floor and the prepontine interval (PPI), which refers to the prepontine subarachnoid space between the tip of the basilar artery and the clivus [1, 19, 22, 24]. The endoscopic approach also allows for minimally invasive biopsy of tumors in the same session if they are accessible through the ventricle [9]. In selected cases where the endoscopic approach was considered risky or not feasible (see the “Methods” section for patient of patient selection criteria), we employed the stereotactic technique. Minimally invasive stereotactic third ventriculostomy has been described as an alternative surgical technique for patients with OH and was associated with low periprocedural morbidity [6]. Although the fenestration at the floor of the third ventricle is smaller than in ETV, persistent patency of the stoma after stereotactic ventriculostomy was reported in 15 out of 16 patients treated for nontumoral aqueductal stenosis [15]. However, a revision rate of 13% most probably due to processes like scar formation, gliosis, and membrane formation was reported in another study [6]. One possible way to overcome this failure rate is to introduce a stereotactic internal shunt catheter which extends through the lateral and third ventricle into the prepontine space. This catheter is fixed at the skull using titanium clips and not connected to an extracranial system [7, 20].

Building upon the experience of low morbidity in placing cystoventricular catheters in patients with cystic craniopharyngioma, which travel through the ventricle into the basal cistern, allowing upstream and downstream drainage of cysts, this stereotactic trajectory has been further applied in selected cases for the treatment of tumor-associated occlusive hydrocephalus [26].

In the current study, stereotactic ventriculostomy with internal shunt placement (sTVIP) was analyzed regarding complication rate, efficacy, and subsequent shunt dependency to assess whether this technique is a feasible alternative for the treatment of tumor-related OH.

## Methods

### Patient selection

All patients with tumor-associated secondary OH who underwent treatment with stereotactic third ventriculostomy including internal shunt placement between January 2010 and December 2021 at our institution were included in the study. Whenever feasible, resection for alleviation of the obstruction or an endoscopic approach was chosen as the primary treatment. sTVIP was preferred for patients with anatomical variations on preoperative MRI, which could complicate the endoscopic approach [25, 29]. Additionally, when biopsy was required and the tumor was not accessible through a transventricular route, the stereotactic procedure allowed simultaneous biopsy and treatment of OH with sTVIP, making it the preferred option. Obstructive hydrocephalus was diagnosed by magnetic resonance imaging including high-resolution T2-weighted and contrast-enhanced T1 sequences. Tumor diagnosis was achieved in all patients either by stereotactic biopsy or resection. The retrospective analysis was approved by the ethics committee of Munich University Medical Center (reference no. 22–0511) and is reported according to institutional guidelines.

### Variables, data sources, and measurement

Eligible patients’ charts and operative notes were reviewed for demographic data, diagnosis, and preoperative clinical status. The main outcome parameters were of symptom improvement, revision-free survival, and complication rate.

The prepontine interval (PPI) was measured on preoperative sagittal T2-weighted MRI sequence as the linear distance between the cortical bone of the dorsum sellae and the basilar artery [34].

### Stereotactic shunt placement technique

**Fig. 1:**
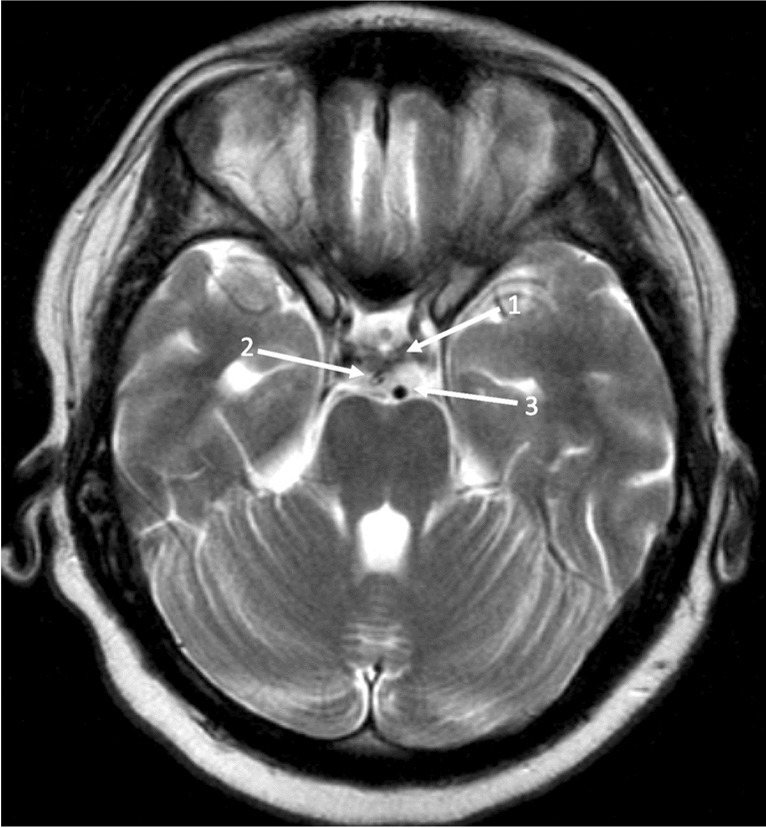
Postoperative MRI of a patient treated with sTVIP. T2-weighted axial MRI showing the cisternal part of the internal shunt (2) posterior to the dorsum sellae (1) and lateral to the basilar artery (3)

**Fig. 2:**
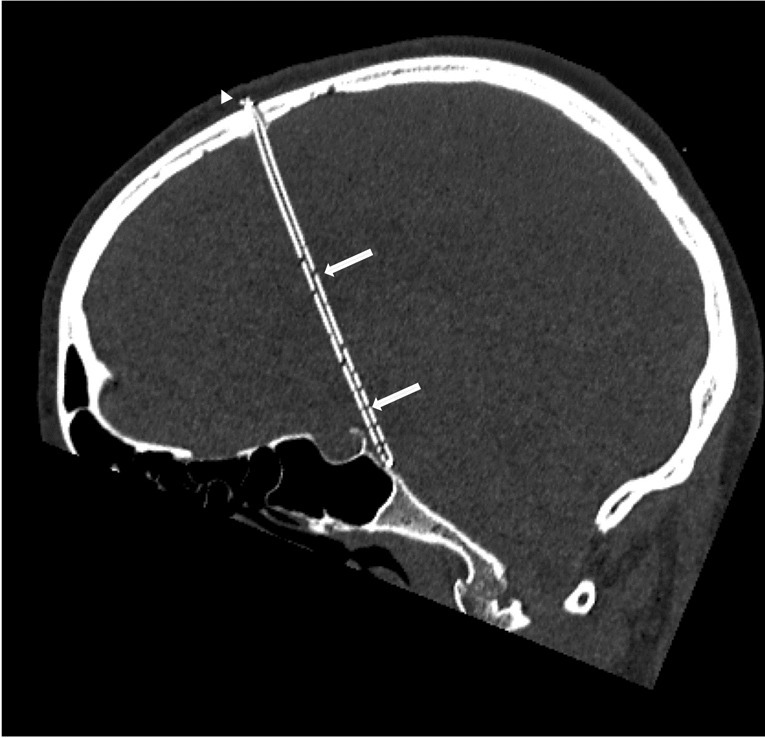
Postoperative CT scan of a patient treated with sTVIP. Bone window in a parasagittal oblique plane showing the entire length of the inner shunt. The catheter is fixed on the skull using a titanium clip (arrowhead) and fitted with appropriate drainage holes on the level of the lateral and third ventricle and the prepontine cistern (arrows)

Patients were operated on under total intravenous anesthesia using propofol and remifentanil. Every surgery was performed using a frame-based multimodal imaging-guided stereotactic technique as previously described [20, 26, 35]. After positioning the stereotactic frame to the patients’ head, a contrast-enhanced computer tomography (CT) was performed and fused to the preoperative acquired contrast-enhanced T1 and T2-weighted magnetic resonance images (MRI) as well as a contrast-enhanced MR angiography. For trajectory planning, a software (Brainlab, Feldkirchen, Germany) allowing triplanar simulation was used. The technical principles of stereotactic shunt placement have been previously described and were modified according to our demands [16, 20, 26]. The target point was located in the prepontine cistern in the subarachnoid space lateral of the basilar artery. The entry point was dependent on the trajectory which passes from the right or left anterior ventricular horn through the foramen of Monro and penetrating the floor of the third ventricle in the midline. After placement of a stereotactic localized twist drill craniostomy and durotomy, the stereotactic cannula was then advanced toward the target point along the calculated trajectory, perforating the third ventricular floor anterior to the mamillary bodies. In an effort to minimize the risk of inadvertent injury to the basilar artery and to avoid hypothalamic injury at the same time, the floor of the third ventricle was perforated in the midline, anterior to the mamillary bodies. Due to the oblique trajectory, the catheter was directed toward a parasagittal target point. This allowed placement of the tip of the catheter in the subarachnoid space lateral to the basilar artery (Fig. 1). After removing the cannula, a vancomycin-impregnated ventricular catheter was inserted under stereotactic guidance using the same trajectory. In order to allow drainage of the third ventricle into the basal cisterns, the catheter was fitted with appropriate drainage holes (Fig. 2, arrows). After insertion, the catheter was fixed on the skull using a titanium clip (Fig. 2, arrowhead). In patients scheduled to undergo simultaneous stereotactic biopsy for histological diagnosis, a separate trajectory was planned. Biopsy of the solid tumor was performed as previously described [14]. Biopsies were always performed before stereotactic shunt placement in order to avoid changes of the anatomy due to changes of ventricle volume, which would make the trajectory planning imprecise. A CT scan confirmed correct placement of the catheter on postoperative day one.

### Statistical methods

The unpaired *t*-test was used to calculate outcome differences of continuous variables, while the Fisher exact test was used for categorial variables. Logistic regression was used to calculate the effect of covariates on binary outcomes. The revision-free survival was analyzed using the Kaplan–Meier method. For all statistical analysis, a *p* value < 0.05 was deemed to be significant. All statistical tests were performed with GraphPad Prism 9 (GraphPad Software, La Jolla, California).

## Results

### Patients’ characteristics (Table 1)

**Table 1:** Patient characteristics

	Value
Age, mean (range)	40 (5–88)
Sex, *n* (%)	
Female	11 (28.9)
Male	27 (71.1)
Histology, *n* (%)	
Pilocytic astrocytoma	5 (13.2)
Astrocytoma WHO grade 1	1 (2.6)
Oligodendroglioma WHO grade 2	0
Astrocytoma WHO grade 3	3 (7.9)
Oligodendroglioma WHO grade 3	1 (2.6)
Glioblastoma	9 (23.7)
Diffuse midline glioma	6 (15.8)
Tectal glioma	3 (7.9)
Brainstem glioma	1 (2.6)
Ependymoma	1 (2.6)
Ganglioglioma	1 (2.6)
DNET	1 (2.6)
Pineocytoma	3 (7.9)
Brain metastasis	3 (7.9)
Presenting symptoms, *n* (%)	
Headache	16 (42.1)
Nausea	7 (18.4)
Decreased vigilant state	4 (10.5)
Diplopia/decreased visual acuity	6 (15.8)
Seizures	4 (10.5)
Focal deficits	7 (18.4)
Asymptomatic	7 (18.4)
Previous treatment, *n* (%)	
None	23 (60.5)
Resection	8 (21.1)
Radiotherapy	5 (13.2)
Brachytherapy	4 (10.5)
Chemotherapy	8 (21.1)

We identified 38 patients with a mean age of 40 years (range 5–88), 11 (28.9%) of them female. Eighteen patients (47.4%) had previously been diagnosed and treated for intracranial tumors. In 20 patients (52.6%), tumor diagnosis was obtained with biopsy at the time of sTVIP. In terms of histological diagnosis, 13.2% (*n* = 5) were diagnosed with pilocytic astrocytoma, 2.6% (*n* = 1) with diffuse astrocytoma CNS WHO grade 2, 7.9% (*n* = 3) with astrocytoma CNS WHO grade 3, 2.6% (*n* = 1) with oligodendroglioma CNS WHO grade 3, 23.7% (*n* = 9) with glioblastoma, 15.8% (*n* = 6) with diffuse midline glioma, 7.9% (*n* = 3) with tectal glioma, 2.6% (*n* = 1) with brainstem glioma, 2.6% (*n* = 1) with ependymoma, 2.6% (*n* = 1) with ganglioglioma, 2.6% (*n* = 1) with DNET, 7.9% (*n* = 3) with pineocytoma, and 7.9% (*n* = 3) with brain metastasis. Tumor locations were thalamic in 36.8% (*n* = 14), lobar in 10.5% (*n* = 4), in the pineal region in 10.5% (*n* = 4), affecting the brainstem in 28.9% (*n* = 11), and multifocal in 13.2% (*n* = 5). 60.5% (*n* = 23) of the patients had not experienced previous treatment, while the other patients had previously undergone resection in 21.1% (*n* = 8), radiotherapy in 13.2% (*n* = 5), and chemotherapy in 21.1% (*n* = 8). Four patients (10.5%) were treated with iodine-125 brachytherapy [31, 33]. The most common preoperative symptom was headache in 42.1% (*n* = 16) of our patients. Other symptoms included nausea and vomiting in 18.4% (*n* = 7), decreased vigilance in 10.5% (*n* = 4), diplopia or reduced visual acuity in 15.8% (*n* = 6), seizures in 10.5% (*n* = 4), and focal neurological deficits (e.g., paresis) in 18.4% (*n* = 7). Seven patients (18.4%) were asymptomatic and diagnosed with OH through imaging studies. Median preoperative Karnofsky index was 80 (range 30–100). Mean time of OH treatment from previous tumor specific treatment was 8 months (range 0–66 months); mean time of OH treatment from the first diagnosis of brain tumor was 17.2 months (range 0–144 months).

### Surgical characteristics

Thirty-eight procedures with stereotactic third ventriculostomy with internal shunt placement were analyzed; in 20 patients (52.6%), concurrent stereotactic biopsy of a tumor mass was performed before sTVIP using a separate trajectory. The average prepontine interval measured on preoperative MRI was 3.2 mm (range 1–5.5 mm). Average surgical times were 44.6 min (± 34.9 min) in the undergoing biopsy in the same procedure and 34 min (± 18.5 min) in the sTVIP only group (*p* = 0.3019). The accuracy of intracranial shunt placement was confirmed by postoperative imaging in all cases. Average hospitalization time was 3.8 days (± 3.11 days) in the group that underwent biopsy in the same procedure and 2.78 days (± 2.98 days) for sTVIP alone (*p* = 0.31).

### Complications

One patient (2.6%) experienced intraventricular hemorrhage which did not cause symptoms but led to revision surgery due to aresorptive hydrocephalus 3 months later. This patient suffered from Gilbert’s syndrome which might have influenced platelet function [18]. Revision surgery was necessary in one patient due to CSF leak (2.6%) occurring 1 month after operation due to obstruction of the catheter. In two patients (5.3%), an asymptomatic parenchymal hemorrhage was detected along the stereotactic trajectory on postoperative CT scan, which did not warrant therapy at any time point. Three patients (7.9%) showed symptoms and radiographic signs of postoperative pneumonia; all three had presented with a decreased vigilant state due to occlusive hydrocephalus so the underlying cause was most probably aspiration.

### Outcome

Postoperative symptom improvement was achieved in 74.2% (*n* = 23) of symptomatic patients (*n* = 31). Headache was improved in 15/16 (94.1%) patients, nausea and vomiting in 6/7 patients (85.7%), vigilance in all patients (*n* = 4), ophthalmologic symptoms in 4/6 patients (66.7%), seizures in 2/4 cases (50%), and focal deficits in 4/8 patients (50%). 39.5% (*n* = 15) remained clinically unchanged, but with regressive radiographic features of OH. Univariate binary logistic regression for postoperative symptomatic improvement was performed for age, gender, presenting symptoms, previous therapy, time from previous therapy to sTVIP, and time from the first diagnosis to sTVIP. Preoperative headache was a significant predictor of postoperative improvement (OR 26.25; 95% CI 4.141–521.1; *p* = 0.004) (Table 2). Postoperative symptom improvement did not predict a longer overall survival (OR 2.42; 95% CI 0.979–6.577; *p* = 0.065).

**Table 2:** Preoperative variables influencing postoperative improvement. Univariate binary logistic regression modeling for symptomatic improvement after treatment with sTVIP. *p < 0.05, **p < 0.01, and ***p < 0.001

Variable	Odds ratio of symptomatic improvement	95% CI	*p*
Age	0.9684	0.9323–1.001	0.0716
Sex			
Female	1.75	0.3735–9.900	0.494
Male	0.5713	0.1010–2.677	0.494
Presenting symptoms			
Headache	26.25	4.141–521.1	0.0036**
Nausea	4.941	0.7236–99.16	0.1607
Decreased vigilant state	7.154	0.357–143.252	0.1385
Diplopia/decreased visual acuity	1.368	0.2305–10.93	0.738
Seizures	0.619	0.06735–5.673	0.6511
Focal deficits	0.8421	0.1580–4.900	0.8394
Previous therapy			
None	4.753	0.1009–355.8	0.4337
Resection	1.645	0.08141–49.51	0.7414
Radiotherapy	3.348	0.1871–142.7	0.4394
Brachytherapy	1.596	0.01587–186.6	0.8374
Chemotherapy	5.515	0.1836–355.7	0.3496
Time to previous therapy	0.9925	0.9379–1.050	0.7811
Time from first diagnosis	1.002	0.9793–1.030	0.8751

**Fig. 3:**
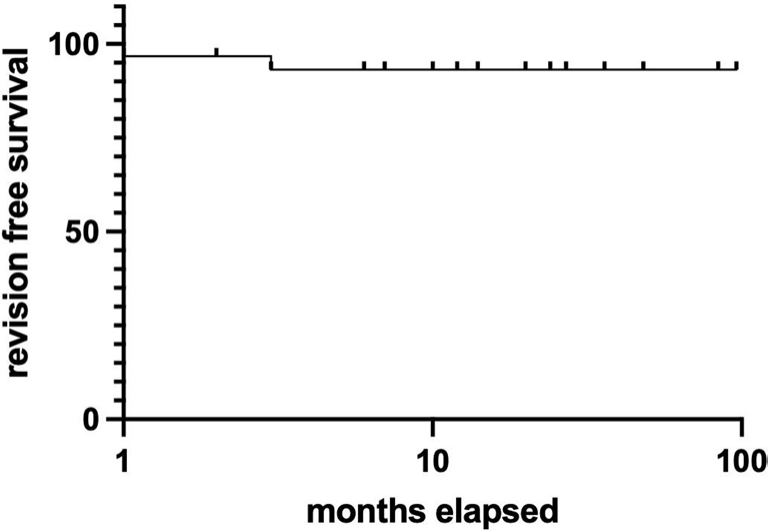
Overall revision-free survival after sTVIP. Kaplan–Meier analysis of the overall revision-free survival for the entire cohort after sTVIP

Revision-free survival was estimated for the entire cohort with the Kaplan–Meier method (Fig. 3). Two patients required revision surgery after 1 and 3 months, respectively. Thirty-one patients were censored due to deaths in 15 patients and loss of follow-up in 16 patients.

### Clinical example

**Fig. 4:**
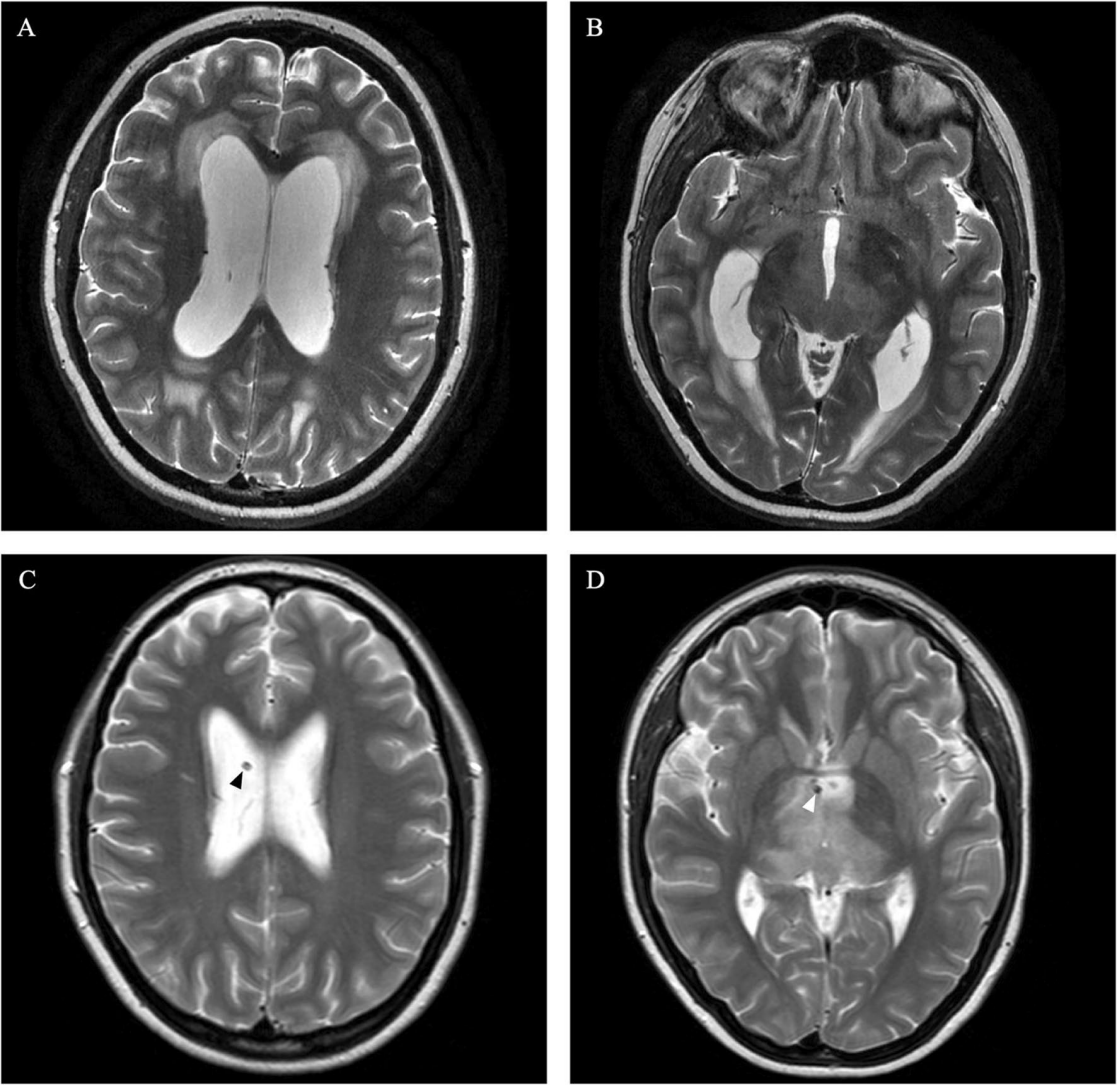
MRI of an illustrative case. T2-weighted axial MRI of patient with obstructive hydrocephalus secondary to a thalamic malignant glioma before sTVIP (**A**, **B**). T2-weighted axial MRI 3 months after inner shunt placement showing reduced dilatation of lateral ventricles indicating patency of the inner shunt (arrowheads, **C**, **D**)

A 26-year-old patient presented to our institution with headache, nausea, vomiting, and gait imbalance. MRI showed a diffuse infiltrating lesion suspicious of a thalamic glioma causing aqueductal stenosis and consecutive OH (Fig. 4A, B). Since the chance of a cure with surgical resection was considered low, the patient was scheduled for stereotactic biopsy followed by sTVIP to treat hydrocephalus in the same procedure. According to clinical standard, the patient was observed for 2 days after surgery and showed improvement of headache and nausea which allowed discharge of the patient. Histological and molecular analysis of the tumor specimen led to diagnosis of a H3K27M-mutated midline glioma. The patient received concomitant radiochemotherapy. At the three-month follow-up, MRI confirmed reduced radiologic signs of hydrocephalus (Fig. 4C, D). The patient still suffered from gait imbalance which was probably caused by the thalamic glioma. Following sTVIP, OH was successfully managed for 11 months until the patient died due to tumor progression.

## Discussion

Obstructive hydrocephalus secondary to intracranial neoplasia has traditionally been treated with ETV and VS. Perioperative morbidity and hospitalization time have been reported to be lower for ETV, hence making it the first choice of treatment [2, 13]. However, in selected patients, we used sTVIP as an alternative treatment for patients with tumor-associated OH and found it to be an efficient alternative to ETV. Considering previous studies from our department demonstrating efficiency and safety of internal shunts for the treatment of arachnoid cysts and cystic craniopharyngioma, we deemed internal shunt placement as a feasible method [20, 26]. Additionally, stereotactic biopsy has already proven to be a safe and effective method allowing histological diagnosis in 96.3% with symptomatic postprocedural complications in 1.2% and no mortality [14, 17]. In some cases of secondary hydrocephalus, the advantage of sTVIP over ETV is that the stereotactic procedure enables the surgeon to obtain tissue specimen even from deep-seated lesions that cannot be reached through the ventricle and therefore are not accessible via a transventricular neuroendoscopic approach.

In the present study, sTVIP was technically successful in all patients as was tissue biopsy, which was performed simultaneously in a little more than half of the cases. The rate of clinical improvement in the current analysis (74.2%) was comparable to rates reported for ETV of 68.5–77.6% [8, 30].

We encountered a CSF leak rate of 2.6% and intraventricular hemorrhage rate of 2.6% after sTVIP, which is comparable to that reported for ETV at 3.1 and 1.7%, respectively [30].

A narrow prepontine interval has been suggested to put the basilar artery at risk during ETV, since the basilar artery shifts anterior to the mammillary bodies [11]. Periprocedural mortality resulting from inadvertent basilar artery injury during ETV is registered in 0.3% [30]. This makes a reduced PPI not a contraindication for ETV, but implicates that special caution has to be used when perforating the floor of the third ventricle [11]. Souweidane et al. (2010) report 15 patients with a PPI ≤ 1 mm that underwent ETV; however, the authors state that “intraoperative stereotactic guidance was heavily relied upon” in cases of diminished PPI [34]. This underlines the fact that stereotactic planning of an operative trajectory is advantageous when dealing with reduced PPI in order to optimize stoma placement and to reduce the risk of intraoperative complications. The prepontine interval in our group ranged from 1 to 5.5 mm (mean 3.2 mm), demonstrating that sTVIP is feasible even in the setting of a nearly obliterated prepontine interval.

Furthermore, in cases of incomplete opening of the Liliequist membrane (LM), positioning of the catheter tip in the prepontine cistern is increasing the chance of perforation of LM with the catheter. Even though LM was not routinely visualized on preoperative MRI, it has been speculated upon as a possible cause of operative failure in ETV and accordingly must be remembered when performing sTVIP [5, 32].

We also believe in sTVIP as a valuable option to endoscopy in patients with thickened third ventricular floor where visualization of the basilar artery is not possible, even though we did not encounter this scenario in the current study [28].

Shunt independency reported for patients treated with ETV varies between 68.5 and 77% [2, 30]. The primary cause of delayed closing of the stoma after ETV is suspected to be obstruction through gliosis or formation of arachnoid membranes [4, 10, 12, 30]. In our study, shunt independence—defined as independence of extracranial CSF diversion—was achieved in 97.4% (*n* = 37) of our patients. Regarding revision-free survival, the high number of censored patients has to be considered (*n* = 31). This is due to progression of malignant glioma causing deaths in 15 patients, 14 of whom passed away within 6 months. The remaining patients were lost to follow-up. Taken together, we cannot estimate if sTVIP would be an adequate option for patients with OH with a long life expectancy. In those patients, ETV carries, among other the advantages, the benefits of not requiring foreign materials with inherent risks of infection and obstruction, direct visualization of the basilar artery intraoperatively, and a long history of studies demonstrating the efficacy and low morbidity of ETV. Based on our data, we can assume that in patients with OH caused by malignant glioma, revision surgery is a rare event making it a valuable option in oncologic patients. Even though in this cohort of 38 patients sTVIP showed low morbidity and no fatal outcomes, further evaluation with larger cohorts is necessary to allow for a better comparison with endoscopic third ventriculostomy.

## Limitations

This study carries all limitations of retrospective studies. This method also has been performed in only a small cohort of patients in a single institution, which makes it influenced by management strategies that are unique to our center. Given the challenges posed by the high number of censored patients, an accurate estimation of the revision-free survival after sTVIP is difficult.

## Conclusions

Stereotactic third ventriculostomy with internal shunt placement is a feasible, safe, and effective method to treat tumor-associated obstructive hydrocephalus in selected patients. In our small retrospective cohort, there was low morbidity and no procedure-related mortality. The stereotactic approach offers the possibility to perform highly precise tissue biopsy in the same surgery, thereby reducing the need for additional surgery, especially in deep-seated locations where biopsy is not possible when using an endoscopic transventricular approach. In an effort to reduce hospitalization and readmission due to shunt failure in patients with a reduced life expectancy, this method seems advantageous for patients with obstructive hydrocephalus secondary to midline high-grade gliomas.

## Data Availability

The data that support the findings of this study are available from the corresponding author, upon reasonable request.

## Abbreviations

sTVIP: Stereotactic third ventriculostomy and inner shunt placement
ETV: Endoscopic third ventriculostomy
VS: Ventriculoperitoneal shunt
OH: Obstructive hydrocephalus
